# Mercury concentration in the feathers of birds from various trophic levels in Fereydunkenar International wetland (Iran)

**DOI:** 10.1007/s10661-016-5671-y

**Published:** 2016-11-12

**Authors:** Mousa Ahmadpour, Li Lan-Hai, Mohsen Ahmadpour, Seyed Hamid Hoseini, Abdolreza Mashrofeh, Łukasz J. Binkowski

**Affiliations:** 1 College of Biology and Environment, Nanjing Forestry University, Longpan Road 159, Nanjing, 210037 China; 2 Department of Environmental Sciences, Faculty of Fisheries and Environmental Sciences, Gorgan University of Agricultural Sciences and Natural Resources, Gorgan, 4913815739 Iran; 3 Department of Environmental Sciences, Faculty of Natural Resources, University of Malayer, Malayer, 6751995863 Iran; 4 Environmental Forensic Laboratory, Department of Environmental Sciences, Faculty of Natural Resource, Tarbiat Modares University, 64414-356, Noor, Iran; 5 Institute of Biology, Pedagogical University of Cracow, Podbrzezie 3, 31-054 Krakow, Poland

**Keywords:** Hg, Wetland, Birds, Pollution, Feather

## Abstract

Mercury (Hg) is one of the main global pollutants that may biomagnify in food nets, especially in wetlands. Birds may be useful in the biomonitoring of Hg in such habitats and may even serve in vivo samples. This paper examined Hg concentration in the feathers of seven bird species foraging on Fereydunkenar International wetland (in 2013). Mean Hg concentrations found ranged from 0.005 ± 0.002 μg g^−1^ d.w. (dry weight) (Common hoopoe) to 0.38 ± 0.047 μg g^−1^ d.w. (Greylag goose). Significant differences in Hg concentrations were noted between bird species as well as between trophic levels (one-way ANOVAs, *p* < 0.001). The decrease in mean Hg concentration in feathers was as follows: Greylag goose > Northern pintail ≥ Gadwall ≥ Mallard > Eurasian bittern ≥ Little bittern > Common hoopoe. The position in the trophic chain significantly influenced Hg concentrations, which were the highest in omnivorous species. Hg concentrations may also depend on migration routes and breeding habitats, but the evaluation of the exposure exceeds the ambit of this paper. The Hg concentrations found generally were low, lower than the safe thresholds reported in the literature.

## Introduction

Mercury (Hg) deposits in the environment continue to be an important environmental issue across the globe. The increase in global Hg emission is still being observed (Pirrone et al. 2010; Streets and Zhang 2009). Hg can be bioaccumulated and biomagnified in aquatic and terrestrial ecosystems, but generally Hg levels tend to be higher in aquatic environments, due to direct runoff or input from rivers, point source pollution, atmospheric deposits, and further accumulation of pollutants in the bottom sediments (Wolfe and Norman 1998; Boening 2000; Driscoll et al. 2007; Cristol et al. 2008; Goodale et al. 2008). This phenomenon leads to wildlife being exposed to it, and this may harm animals and even lead to population declines (Burger and Gochfeld 2009).

Wetlands combine aquatic and terrestrial ecosystems. They are inhabited by numerous groups of animals, of which waterfowl is one of the most abundant. The Hg effects on birds may vary, but mainly include lethargy and the disruption in the endocrine system as well as a change in mating and parenting behavior (Jayasena et al. 2011; Heath and Frederick 2005; Evers et al. 2008; Frederick and Jayasena 2010; Hallinger et al. 2010). The population decline in several of waterfowl species has led to an increase in attempts to study the Hg concentration in waterfowl in the wild as well as the effects of Hg on their population and physiology (Monteiro and Furness 1995; Provencher et al. 2014; Wayland et al. 2008). Hg exposure in waterfowl is a multi-step process that involves direct uptake through ingestion, transportation in blood, and subsequent accumulation in internal tissues such as the liver, kidneys, and muscle tissue (Binkowski et al. 2016). Hg elimination is possible via deposit in eggs, excreta, uropygial gland, salt gland, and feathers (Burger 1993; Dauwe et al. 2000). Redistribution to plumage (material of the highest Hg density) occurs during feather growth. This material is useful for measuring Hg contamination and exposure because it may be examined without killing or even capturing the animal (Karimi et al. 2016). Levels in feathers reflect blood levels during the short period of feather growth, when the feather is connected to blood vessels, and metals are incorporated in the keratin structure. Sulfhydryl groups of keratin form strong bonds to metals, so keratin is even treated as the chelating agent. When the feather matures (usually 1–3 weeks), blood vessels shrivel and the feather is no longer supplied with blood, at which point the metal deposition to feather ceases. (Burger 1993). The proportion of the burden in the body and the feathers is relatively constant for each metal (Burger and Gochfeld 2000a; Burger and Gochfeld 2000b). The highest proportions were observed for Hg, reaching up to 90% of the body burden in feathers with concentrations found up to 170 μg/g d.w. (Burger 1993). Depending on sample preparation prior to analysis, feathers may also be used to evaluate external contamination through dust and particulate matter.

In Fereydunkenar International wetland (FIW), recent applications of fertilizers and pesticides by farmers have resulted in increased accumulation of metals, including Hg in water and soil. Thus, the transfer from the environment into the animals in the area is a real possibility (Mashroofeh et al. 2015). Hg levels in local bird species (e.g., Common hoopoe) may serve the image of this exposure. Migratory waterfowl species, as studied here, also reflect the degree of pollution in stopover and breeding sites in Russia and Eastern Europe (Ahmadpour et al. 2012). Since the local people in FIW are prolific hunters of migratory birds, this study also provides comprehensive information regarding human exposure to Hg.

The main aim of the study was to investigate the total Hg concentrations in the primary feathers of seven bird species: Greylag goose, Northern pintail, Gadwall, Mallard, Eurasian bittern, Little bittern and Common hoopoe, representing various trophic levels in FIW. The differences between species and the differences between the trophic levels were evaluated.

## Materials and methods

### Study area

FIW is composed of Fereydunkenar, Sorkhrud, and Azbaran lagoons which were registered in the Ramsar Convention on Wetlands. The area is located to the southern Caspian Sea at the coordinates of 52° 35′ to 52° 25′ E and 36° 35′ to 36° 45′ N. The ecosystem is a woody-type wetland which covers 5427 ha, contains important habitats and attracts about one third of Iran’s bird species (Ahmadpour et al. 2012). In this area, farmers use chemical fertilizers and pesticides, especially fungicides in the cultivation of rice.

### Collection of feathers and preparation for analysis

Feathers were taken from 24 birds (collected by hunters) from seven species in FIW between October and December 2013 (Table 1). The following bird species were studied: Greylag goose (*Anser anser*), Northern pintail (*Anas acuta*), Gadwall (*Anas strepera*), Mallard (*Anas platyrhynchos*), Eurasian bittern (*Botaurus stellaris*), Little bittern (*Ixobrychus minutus*), and Common hoopoe (*Upupa epops*).

**Table 1:** Hg concentrations (μg g^−1^ d.w.) in feather samples of birds studied from FIW

Family	Trophic level	Common name	*N*	Mean (d.w.) ± SD	Mean (w.w.^*^) ± SD
Anatidae	^#^Omnivore	^a^Greylag goose	3	0.380 ± 0.047	0.333 ± 0.041
		^ab^Northern pintail	3	0.280 ± 0.040	0.246 ± 0.035
		^ab^Gadwall	4	0.380 ± 0.048	0.333 ± 0.042
		^ab^Mallard	5	0.280 ± 0.050	0.246 ± 0.044
Ardeidae	^&^Piscivore	^bc^Eurasian bittern	3	0.170 ± 0.049	0.149 ± 0.043
		^cd^Little bittern	3	0.110 ± 0.060	0.096 ± 0.053
Upupidae	^§^Insectivore	^d^Common hoopoe	3	0.005 ± 0.002	0.004 ± 0.002

Different letters and signs indicate significant differences (one-way ANOVA followed by the Tukey test)

^*^Concentrations given in w.w. were recalculated according to data and protocols described in the literature (Binkowski 2012; Binkowski and Sawicka-Kapusta 2015)

The two inner and two outer primary feathers of the right wing were collected from each bird, placed in labeled envelopes, and stored in a light-inhibiting box until they were transported to the environmental laboratory for analysis (Faculty of Natural Resources and Marine Sciences, Tarbiat Modares University). The feathers were then washed using tap water, rinsed three times alternating between ultrapure water (RODI RO2000 system) and acetone (Fluka capillary GC grade 99.9%) to remove external contamination, and dried out in an oven at 60 °C (Memmert BE 500) for 24 h. Finally, they were cut into approximately 1-mm pieces and stored in desiccators until they were analyzed chemically.

### Chemical analyses of samples

Between 50 mg and 100 mg of the homogenized powder of dried sample was added to 8 mL of nitric acid HNO_3_ (Merck 65% supra-pure, Darmstadt, Germany) in closed polytetrafluoroethylene (Teflon™) lined digestion vessels and incubated for 1 h at 40 °C. The temperature was then increased to 100 °C for 2 h. Samples were left to cool. Then, 2 mL of H_2_O_2_ (Merck 30% ISO grade Darmstadt, Germany) was added, and the sample was heated again until any precipitation was fully dissolved. Upon cooling, 5% potassium permanganate (KMnO_4_, ACS reagent) was added to ensure oxidation of all organic Hg compounds. The samples were then heated again to 90 °C for 30 min, cooled, moved to volumetric tubes, where hydroxylamine hydrochloride was added (Fluka, AAS grade, 99.9%) to reduce excess oxidizing reagents, and were diluted with ultrapure water to 25 mL. All the mineralization stages were carried out in Milestone START D Microwave Digestion System.

The elemental Hg concentrations were measured by a cold vapor atomic absorption spectrometer (PerkinElmer AA 700). Argon (Arkan gas, grade 5.0) was used as the carrier gas. Standard solutions (100, 200, and 300 ppb) were prepared with 1000 ppm standard Hg solution (Fluka, analytical standard solution), HNO_3_ (to achieve 1.5% weight in volume (*w*/*v*) of standard solution), 5% KMnO_4_ (to fix the solutions), and a mixture of caustic soda and sodium boron hydrate solution (to achieve 1 and 3% *w*/*v* of standard solution, as regenerative and to react in the reaction flask and release Hg vapors from the samples). The parameters obtained for the calibration curve were good (linearity 0.9996; standard error of the estimate 0.0046).

Final Hg concentrations are expressed as micrograms per gram d.w. (dry weight), but they were also recalculated to give their values in w.w. (wet weight). The quality assurance and quality control procedures were assessed using control standard solutions and spikes. The recoveries all ranged from 88.6 to 99.2%. Each sample was analyzed three times, and the relative standard deviation (RSD) between them was calculated. If the RSD was lower than 15%, the mean of replicates was used. Otherwise, the sample was reanalyzed.

### Statistical analyses

The statistical analyses were all carried out using R programming (R version 3.1.2). The data fitted the assumption of parametric tests (checked using the Shapiro–Wilk and Levene tests), so the one-way ANOVA followed by the Tukey test was used to evaluate the effects of species and trophic level on Hg concentrations in feathers. The significance level was set at 0.05 value in all the analyses.

## Results

**Fig. 1:**
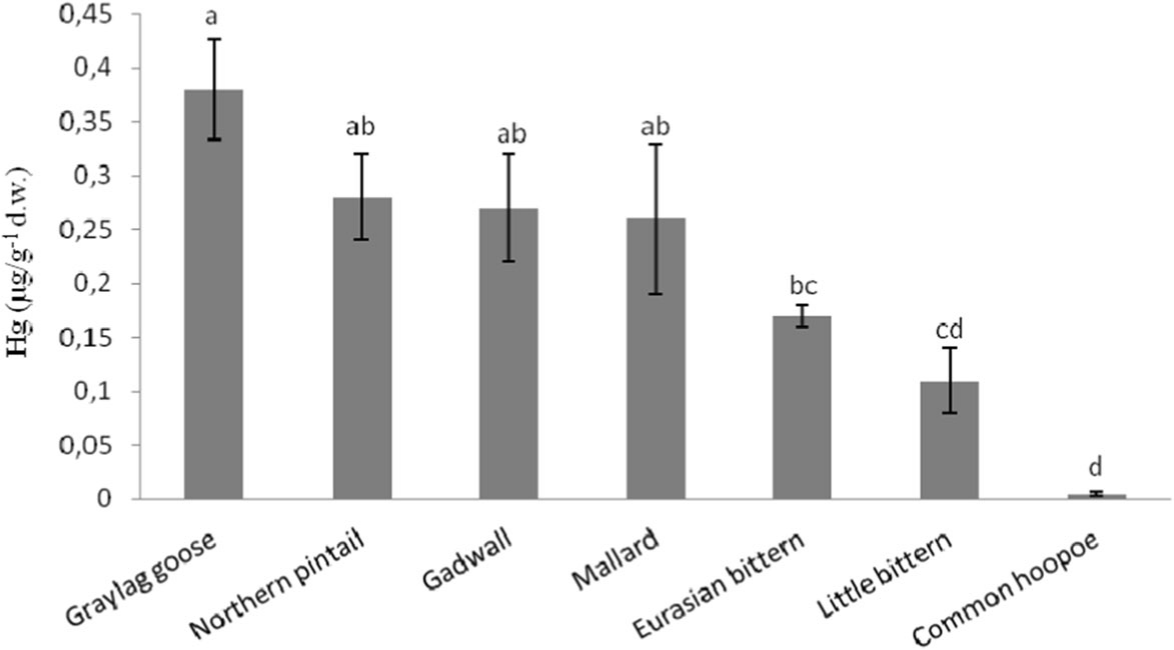
Comparison of Hg concentrations in feathers (mean ± SD) between the species studied. Different *letters* indicate statistically significant differences (one-way ANOVA)

**Fig. 2:**
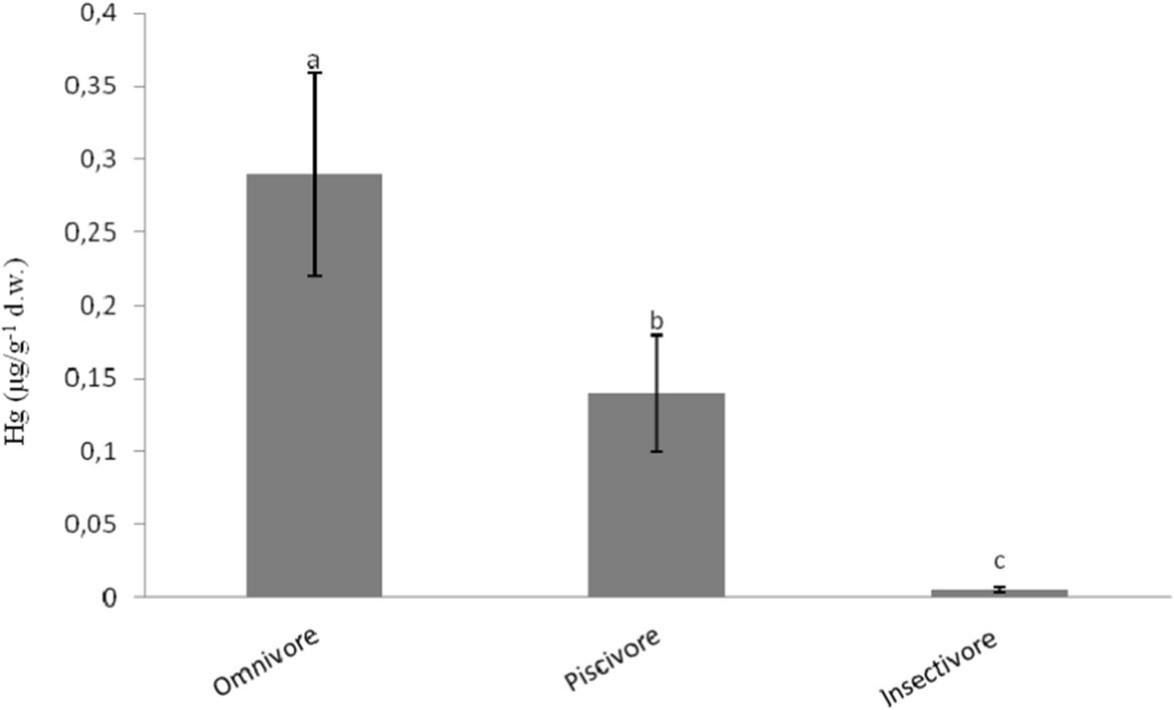
The effect of the trophic level on the comparison of Hg concentrations in feathers (mean ± SD). Different *letters* indicate statistically significant differences (one-way ANOVA)

Hg concentrations in the feathers of birds from FIW ranged from 0.005 ± 0.002 μg g^−1^ d.w. (Common hoopoe) to 0.380 ± 0.047 μg g^−1^ d.w. (Greylag goose; Table 1). In general, the average Hg concentration in the feathers from the highest to lowest value was as follows: Greylag goose > Northern pintail ≥ Gadwall ≥ Mallard > Eurasian bittern ≥ Little bittern > Common hoopoe (Fig. 1). There was a significant difference between these species (*F*_6, 17_ = 19.654, *p* < 0.001). The Greylag goose had significantly higher Hg levels in their feathers than did the other species. Differences in Hg concentrations in feathers were also tested according to the trophic level according to the dietary habits of the birds: omnivorous, piscivorous, and insectivorous. The mean Hg concentrations in these three trophic levels decreased in the following order: omnivorous > piscivorous > insectivorous (*F*_2, 21_ = 36.491, *p* < 0.001, Fig. 2).

## Discussion

We found that the Hg concentrations in our study varied significantly between the species. The highest value was noted in the Greylag goose, and the lowest was found in the Common hoopoe. This variation is said to occur due to differences in body size, physiology, metabolic rates, and activity (Bearhop et al. 2000; Becker et al. 2002). As our study shows, the diet and its derivative, the trophic level, also significantly influence Hg concentrations.

The feather is a discrete depuration and sequestration repository for Hg during the period of feather growth (Burger et al. 2011). When molting is completed, the level of Hg in the feather remains stable, even if the bird feeds on contaminated food. The increase of Hg concentrations is then noted in internal organs (Dauwe et al. 2003). Hg content in feathers represents up to 93% of the accumulated body burden (Bearhop et al. 2000). The molting pattern, migration strategies, and different migration stopovers may convolute the interpretation and utility of the total Hg concentrations in feathers as an indicator of Hg exposure in the sampling area (Furness et al. 1986; Thompson and Furness 1989).

All the birds in the study have a slow rate of molting. Bitterns molt in winter, whereas the Greylag goose, Northern pintail, Gadwall, and Mallard molt in summer. Because of that, the Anseriformes studied reflect the Hg exposure on their breeding habitat, Russia and Eastern Europe where agricultural production such as sugar beet, maize, and cereals is common (Ahmadpour et al. 2012; Kear 2005; Table 2). Evaluating the exposure over such an extensive area is impossible here. The best way to carry out such an evaluation is to use the data for Hg concentrations in ringed and tracked birds. In contrary, bitterns and especially the Common hoopoe reflect the Hg exposure on or near FIW area. Hg intake may reduce appetite leading to weight loss, progressive weakness with lack of coordination, and difficulty in flying, walking, and standing. Hg feather levels between 5 and 65 μg g^−1^ d.w. are also associated with sublethal and reproductive effects (Burger and Gochfeld 1997). In the present study, the Hg levels in the feathers of all the birds were significantly below these thresholds. Generally, the concentrations found were low. The mean Hg concentration (0.21 μg g^−1^ d.w.) was significantly lower than the values reported in feathers of 180 species from various parts of the world (5.6 μg g^−1^ d.w.) (Burger 1993), including 18 species from southwestern Iran (0.87 μg g^−1^ d.w.) (Zolfaghari et al. 2007), Chilean birds (1.7 μg g^−1^ d.w.) (Ochoa-Acuña et al. 2002), 18 species from southern Georgia (0.36 μg g^−1^ d.w.) (Becker et al. 2002), and 12 species from Midway Atoll (3.53 μg g^−1^ d.w.) (Burger and Gochfeld 2000c).

**Table 2:** Potential breeding and wintering sites of species studied

Species	Breeding areas	Wintering areas
Greylag goose	Across Europe from Iceland and UK to northern Russia, Poland, Slovakia, eastern Hungary, and Romania. In Asia broad swathes of the continent as far as China.	From Europe, birds migrate southwards to the Mediterranean and North Africa. From Asia to Baluchistan, Azerbaijan, Iran, Pakistan, northern India, Bangladesh, and China.
Northern pintail	Northern areas of Eurasia and as far south as Poland and Mongolia.	Northern sub-Saharan Africa and tropical South Asia.
Gadwall	Northern areas of Europe and Asia.	South of its breeding range.
Mallard	Across Eurasia, from Iceland and southern Greenland and Morocco in the west, Scandinavia in the north, and to Siberia, Japan, and South Korea, in the east.	South of its breeding range.
Eurasian bittern	Temperate parts of Europe and Asia from the British Isles, Sweden, and Finland eastwards to Sakhalin Island in eastern Siberia and Hokkaido Island in Japan.	The Mediterranean Sea, the Black Sea, Iran, Afghanistan, Kazakhstan, Mongolia, and Hebei Province in northern China.
Little bittern	A range wider than that of the Eurasian bittern (especially towards western parts of Europe and southern parts of Asia).	South of its breeding range.
Common hoopoe	Across Europe and Asia.	South of its breeding range.

On the basis of data in the literature (Madge and Burn 1992; Reichlin et al. 2009; Svenson 2008; Voisin 2010)

Hg concentrations in omnivorous species were higher than in piscivorous birds, which we had not initially suspected. Piscivorous birds show a strong preference for feeding along coasts, in lowlands, and in marshes around lakes, where Hg concentrations are high. In general, the highest Hg accumulation is observed in the most pelagic-oriented fish species and the lowest accumulation is usually in benthic-oriented species (Eagles-Smith and Ackerman 2014). In contrast, Hg concentrations in freshwater fish from bodies of water such as rivers are consistently low. However, the particular wetland site and habitat type may substantially influence fish Hg concentrations. The time of year is also instrumental in the total Hg concentrations in fish where Hg concentrations in fish increased in spring and reached a maximum value in June, before decreasing again in fall (Eagles-Smith and Ackerman 2009). This may result in lower Hg concentrations in the bitterns studied, as they molt at times of relatively low Hg concentrations in fish. The slightly lower concentrations in Little bitterns may be due to their smaller size and thus smaller general intake of food than Eurasian bitterns (Burger 2002). Additionally, a smaller body size implies a faster metabolic rate, which may cause rapid Hg turnover, including accumulation and excretion. A similar mechanism has been observed between the Greylag goose and other Anseriformes studied. Concentrations in omnivorous species are less time dependent than in piscivorous species. In this study, the lowest Hg concentration was noted in the Common hoopoe, whose diet consists mainly of insects, particularly larvae, pupae, and short grass. Additionally, only this species is inextricably linked with the FIW habitat and reflects the exposure to Hg in the area. Based on that fact, we conclude that in FIW, exposure is relatively low. It should be pointed out that the Common hoopoe was the only insectivorous we studied. This species is of course fully insectivorous, but when there is only one species in the group tested, we can make no definitive inferences about the accumulation in insectivorous birds, since we are not able to verify the species based variation. To resolve this limitation, we shall include more species in the group in the future projects.

## Conclusion

Hg concentrations found were not high. They varied between species and between trophic levels of the species studied. Concentrations found in Anseriformes, which accumulated the highest amount, reflected exposure at their breeding sites (mainly Russia and Eastern Europe). Bitterns molt in winter, so the Hg burden in their feather reflected the exposure in wintering areas. Only concentrations found in the feathers of the Common hoopoe reflected the exposure in FIW. On this basis, we conclude that FIW is not contaminated with Hg.
